# The reciprocal interactions between microglia and T cells in Parkinson’s disease: a double-edged sword

**DOI:** 10.1186/s12974-023-02723-y

**Published:** 2023-02-12

**Authors:** Yuxiang Xu, Yongjie Li, Changqing Wang, Tingting Han, Haixuan Liu, Lin Sun, Jun Hong, Makoto Hashimoto, Jianshe Wei

**Affiliations:** 1 Institute for Brain Sciences Research, School of Life Sciences, Henan University, Kaifeng, 475004 China; 2 Henan International Joint Laboratory for Nuclear Protein Regulation, Henan Medical School, Henan University, Kaifeng, 475004 China; 3 Department of Rehabilitation Medicine, Beijing Jishuitan Hospital Guizhou Hospital, Guizhou Provincial Orthopedics Hospital, Guiyang, China; 4 Henan Key Laboratory of Polyoxometalate Chemistry, College of Chemistry and Chemical Engineering, Henan University, Kaifeng, 475004 Henan China; 5 Tokyo Metropolitan Institute of Medical Science, Tokyo, 156-8506 Japan

**Keywords:** Microglia, T cell, Cell–cell interactions, Chemokines, Parkinson’s disease, Cytokines

## Abstract

In Parkinson's disease (PD), neurotoxic microglia, Th1 cells, and Th17 cells are overactivated. Overactivation of these immune cells exacerbates the disease process and leads to the pathological development of pro-inflammatory cytokines, chemokines, and contact-killing compounds, causing the loss of dopaminergic neurons. So far, we have mainly focused on the role of the specific class of immune cells in PD while neglecting the impact of interactions among immune cells on the disease. Therefore, this review demonstrates the reciprocal interplays between microglia and T cells and the associated subpopulations through cytokine and chemokine production that impair and/or protect the pathological process of PD. Furthermore, potential targets and models of PD neuroinflammation are highlighted to provide the new ideas/directions for future research.

## Introduction

Parkinson's disease (PD) is the second most prevalent neurodegenerative disease after Alzheimer's disease and is characterized by the loss of dopaminergic neurons in the substantia nigra, which leads to the reduction of dopamine influx in the nigrostriatal pathway and the appearance of Lewy bodies in the nerves and axons [1, 2]. PD patients may exhibit a range of motor symptoms (e.g., resting tremor, bradykinesia, shuffling gait, dystonia) and non-motor symptoms (e.g., cognitive impairment, anxiety, autonomic dysfunction, sleep disorders) [2, 3]. Epidemiological studies of PD have shown that the characteristics of PD are independently associated with age and gender, and age is one of the essential factors in its development [4]. A report of an epidemiological study published in 2018 on a North American population of patients with PD showed that the prevalence ranged from less than 1% of patients between 45 and 54 years old [5, 6]. In comparison, the number of patients aged 85 years was 4%, and the number of male patients was twice as high as that of female patients [4–8]. The number of PD patients is expected to increase by more than 50% by 2030 due to the aging of the population and increasing life expectancy. Therefore, it is urgent to determine how to effectively treat PD [9].

Recent studies suggest that PD is associated with a variety of factors, including family genetics [10], disorders of the gut microbiota [11], pathogenic infections [12], air pollution [13, 14], head trauma [15], making the understanding of the exact pathogenesis of PD more challenging. The pathogenesis of PD has been explored from various perspectives, including epidemiology, neuropathology, proteomics, genomics, and immunology, and possible mechanisms that cause PD were proposed, such as mitochondrial dysfunction [16–18], oxidative stress [17–20], protein aggregation [21, 22], and abnormal autophagy [23, 24]. Among them, immunological studies suggest that PD results from an imbalance in the immune system homeostasis in pathological processes in PD patients and related animal models [25–27]. Compared to controls, immune cells infiltrate the brain parenchyma and peripheral tissues, causing a release of large amounts of inflammatory and regulatory factors [26, 27]. Furthermore, the activation of pre-existing immune cells in the brain also promotes the damage of dopaminergic neurons and exacerbates the progression of the disease [8, 28]. These findings further reveal the possible mechanisms of PD pathogenesis from an immunological perspective.

Currently, most studies focus on the role of a single cell (microglia, T cells and their subtypes) in the pathological process of PD, which helps researchers to clearly articulate the specific roles of these cells in the pathological process and associated mechanisms of PD. However, various immune cells with mutual regulatory influences are required to prevent the onset of the disease. As a result, studying the reciprocal cooperative regulation of microglia and T cells in PD is necessary. Current reports show minocycline reduces the inflammatory response and decreases microglia proliferation and IL-1β production [29–31]. However, the effect of minocycline on T cells has a different phenomenon between species [32, 33]. Treatment with minocycline in rodents had no impact on T cells and IFN-γ production [32]. Still, human-derived T cells treated with minocycline reduced the proliferation of cells and the ability to release pro-inflammatory cytokines [31, 33]. In addition, minocycline treatment reduced inflammation by decreasing the expression of adhesion molecules and reducing the interaction between T cells and microglia [33]. Notably, increased IL-10 production was only found in microglia and T-cell co-cultures after higher concentrations of minocycline treatment [33]. However, one of the possible reasons for the failure of minocycline to effectively treat amyotrophic lateral sclerosis in the clinic is that its concentration did not reach the concentration used in in vitro cellular assays [34]. It suggests that the interactions between immune cells are essential in treating various diseases. In particular, the phenomenon of interactions between microglia and T cells in PD was previously demonstrated and played a vital role in the pathological changes of the disease [35, 36].

Therefore, this review demonstrates the impact of the reciprocal effects between T cells and microglia in PD on the pathological process of PD through cytokine and chemokine production. Also, potential targets and models in PD neuroinflammation are highlighted to provide new ideas/directions for future research.

## Role of microglia and related mechanisms in PD

Microglia are macrophages residing in the central nervous system (CNS). As critical immune cells in the brain, they perform an irreplaceable role in brain health. Under normal physiological conditions, microglia are in a relatively resting state, monitoring the brain microenvironment and protecting the brain homeostasis by secreting neurotrophic factors to the corresponding neurons [28, 37, 38]. First, microglia can remove invading pathogens, abnormal metabolic cells, and protein fragments from the brain through phagocytosis, thus actively removing potential threats to brain homeostasis [28, 39]. Concomitantly, altered microglia further remove potentially threatening substances by producing cytokines and, chemokines, and through other ways.

It is believed that microglia have different cellular phenotypic profiles, which may be influenced by the local microenvironment and other factors [40, 41]. Microglia have a complex "sensome" for sensing changes in the brain environment [40]. Alterations in the microglia's epigenome, transcriptome, proteome, and metabolome can affect their morphology, ultrastructure, or cellular function [40, 42]. The state of microglia is associated with their unique functions, and the clearance of potentially threatening substances from the brain by microglia involves the interrelation of different states of microglia [40].

**Fig. 1:**
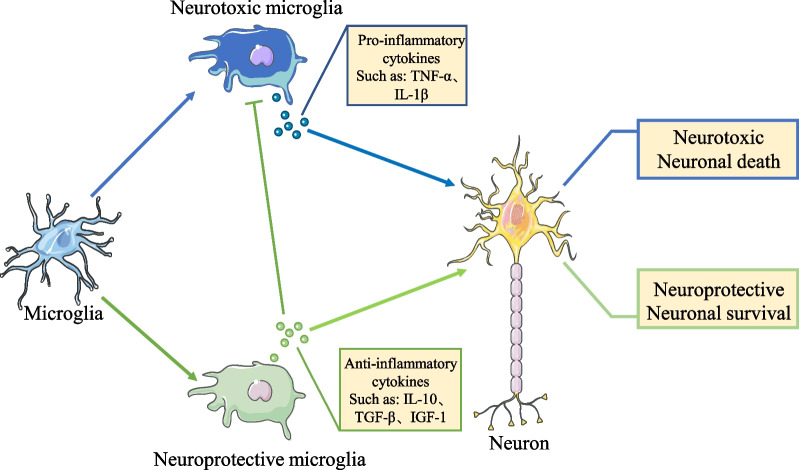
Role of microglia on neurons in Parkinson’s disease (PD). The role of microglia in PD may be neuroprotective and neurotoxic, and their cellular state is altered depending on the external environment. In PD, neurotoxic microglia are hyperactivated and increase inflammation in the microenvironment by releasing pro-inflammatory cytokines that are toxic to neuronal cells and lead to their death. Conversely, anti-inflammatory cytokines released by neuroprotective microglia reduce the number and function of neurotoxic microglia. In addition, neuroprotective microglia interact with neurons to protect neuronal survival, thereby alleviating neuronal death caused by the storm of inflammatory factors in PD

In the PD environment, microglia may be neuroprotective and neurotoxic, and the balance between these changes depends on time and environmental changes [43–45]. When microglia recognize a potentially threatening substance, their cellular state changes, and they kill the threatening substance by releasing pro-inflammatory cytokines and chemokines. In contrast, the excessive release of inflammatory factors causes neurotoxicity, described as "neurotoxic microglia". Moreover, microglia regulate the function of neurotoxic microglia by releasing anti-inflammatory cytokines and chemokines, thus restoring the homeostasis of the brain microenvironment, described as "neuroprotective microglia" [46, 47] (Fig. 1). However, the hyperactivation of microglia in PD may be caused by multiple factors. In vitro, cellular experiments revealed that aberrant α-synuclein in PD mediates the transition of microglia to a neurotoxic state through TLR2, TLR4-NK-κB, and LC3-related phagocytosis, thus exhibiting increased phagocytosis and production of pro-inflammatory cytokines [48–52]. In addition, impaired autophagy of microglia in PD resulted in increased intracellular NLRP3 (NOD-like receptor family, pyrin domain containing 3) activity, which also facilitated the transition to a neurotoxic state in microglia [53]. Co-culture of neurotoxic microglia with dopaminergic neurons promoted the death of dopaminergic neurons [54]. In contrast, culture of neurotoxic microglia with neuroprotective microglia and then co-culturing the fraction with dopaminergic neurons reversed the phenomenon of dopaminergic neuron death caused by neurotoxic microglia [54]. This may be due to the pro-inflammatory cytokines (IL-1β, TNF-α, chemokines) released by overactivated neurotoxic microglia that alter the brain homeostasis, making it more favorable for microglia conversion to neurotoxic, thus causing neurotoxic microglia proliferation and release of inflammatory cytokines. In addition, TNF-α released from neurotoxic microglia can directly induce apoptosis by binding to the tumor necrosis factor receptor-1 (TNFR1) of dopaminergic neurons, a process that may be related to the inhibition of c-Rel with anti-apoptotic function by tumor necrosis factor-alpha (TNF-α) in dopaminergic neurons [55]. C-Rel, one of the five DNA-binding proteins that make up the nuclear factor-kappa B (NF-κB) complex, and its competition with Rel A can downregulate the transcription of apoptotic genes such as Bim and Noxa while initiating the transcription of the anti-apoptotic gene Bcl-2 in cells to maintain neuronal survival [56–59]. In addition to causing direct neuronal damage, neurotoxic microglia can amplify inflammatory phenomena in the PD brain by altering astrocyte status [52, 60]. In the physiological state, astrocytes protect neuronal survival by secreting neurotrophic factors required by neurons. In contrast, neurotoxic microglia in PD cause further neuronal damage by releasing pro-inflammatory cytokines that convert astrocyte phenotype to the neurotoxic A1 type [52, 60].

There are three possible reasons for the emergence of the phenomenon of microglia hyperactivation: (1) Microglia mitochondrial dysfunction. Normal mitochondria via tunneling nanotubes transfer into microglia with abnormal mitochondrial function, the oxidative stress levels and the release of pro-inflammatory cytokines in microglia were downregulated, and the dysfunction of microglia was restored, which in turn alleviated the loss of dopaminergic neurons in PD [61]. (2) The ratio of neurotoxic microglia to neuroprotective microglia is imbalanced. Inhibiting the expression of Jmjd3 in the mouse midbrain impaired the conversion of microglia to neuroprotective microglia and increased dopaminergic neuronal damage caused by the overactivation of neurotoxic microglia [62]. Furthermore, the transfer of in vitro induced differentiation of neuroprotective microglia to mice at different time points of the disease effectively reduced the inflammatory state in the CNS, thereby protecting neurons [63]. (3) Peripheral immune cell infiltration. Overactivation of neurotoxic microglia causes an increase in the level of pro-inflammatory factors, and microglia remove potentially threatening substances while altering the permeability of the blood–brain barrier (BBB), causing infiltration of peripheral immune cells, which also contributes to the overactivation of neurotoxic microglia [64, 65].

Neuroprotective microglia are a group of immune cells with the ability to regulate the inflammatory state by releasing corresponding protein molecules (arginase-1, chitinase 3-like 3, cluster of differentiation [CD] 206) and cytokines (insulin-like growth factor-1, transforming growth factor-beta [TGF-β], interleukin [IL] -10) as well as phagocytosis to remove cellular debris from the internal environment, promote tissue repair, and regulate neuronal damage caused by the overactivation of neurotoxic microglia in the brain (Fig. 1). It is instrumental in maintaining the brain microenvironment homeostasis [66–68]. However, in PD, persistent stimulation of microglia by endogenous factors leads to a reduced conversion of microglia to a neuroprotective state and, thus, to a reduced ability to regulate inflammation, which in turn leads to hyperinflammatory phenomena [69]. The reason for this phenomenon may be the continuous proliferation of neurotoxic microglia in PD and the corresponding release of inflammatory cytokines that maintains the brain microenvironment inflamed. At this time, the relevant factors that promote the conversion of neuroprotective microglia are not secreted sufficiently, thus reducing the conversion of microglia to neuroprotective microglia. In addition, due to the increased oxidative stress in the brain environment of PD patients and animals, Rel A is continuously activated in microglia that respond to oxidative stress, resulting in the continuous activation of the NF-κB pathway that facilitates the conversion of microglia to neurotoxic microglia [57, 58, 70]. However, pathways such as NF-κB/C-Rel, which induce microglial to neuroprotective microglia conversion, are inhibited [67].

## The role of T cells and related mechanisms in PD

**Fig. 2:**
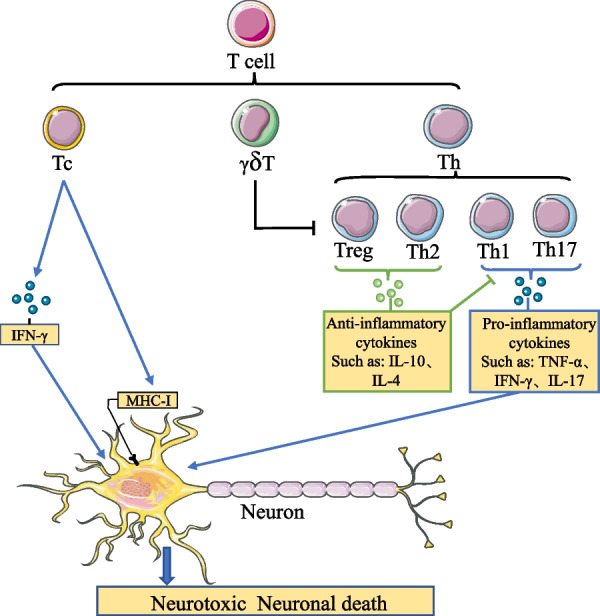
The role of peripherally infiltrating T cells on neurons in PD. Since the damage of the BBB in PD allows T cells in the peripheral circulation to infiltrate the brain, Tc cells infiltrating the brain can recognize MHC-I molecules expressed on the surface of neurons and release granzyme and perforin, to directly cause neuronal death. In addition, Tc cells can also cause neuronal death by releasing IFN-γ. The Th cells infiltrating the brain can be divided into pro-inflammatory Th1 and Th17 and anti-inflammatory Th2 and Treg. Pro-inflammatory Th cells can cause neuronal death by releasing inflammatory cytokines and exacerbating cytokine storms. In contrast, anti-inflammatory Th cells can protect neurons by reducing the release of inflammatory cytokines through the release of anti-inflammatory cytokines, thereby regulating the activity and function of pro-inflammatory Th cells. However, it is worth noting that γδ T inhibits the activity and function of Treg cells and thus reduces their ability to regulate the inflammation

T cells are one of the most important adaptive immune cells in the body, showing powerful functions in cleaning up abnormal cells, invading foreign pathogens, and playing an irreplaceable role in maintaining health. Abnormalities in T-cell activation and function are closely associated with the development of many diseases, for example, tumors, infections, and cardiovascular diseases. In recent years, T cells have also been found to play an essential role in neurodegenerative diseases. Post-mortem examinations of PD patients and some animal models revealed significant T-cell infiltration in the PD brain, altered surface characteristics of T cells in the peripheral circulation, and a marked reduction in cell numbers. This indicates that T cells actively respond to and participate in the onset and progression of PD. The infiltrated T cells in PD brains were classified according to their cell surface markers, and they were distinguished into two major types, CD4^+^ T cells and CD8^+^ T cells. Further studies focus on the two types of T cells function in PD. It reveals that both types of T cells infiltrate the substantia nigra of the PD brain, with CD8^+^ T cells being the main type, which was activated by recognizing target cells expressing major histocompatibility complex (MHC)-I molecules and releasing lymphotoxins such as perforin and granzyme to directly kill the target cells [71, 72] (Fig. 2).

Meanwhile, other studies targeting T cells in PD have found that CD4^+^ T cells play an equally important role in the onset and progression of the disease [73, 74]. Increased motor dysfunction in PD patients was associated with a rise in the number of effector memory CD4^+^ T cells [75]. In addition, the study reveals the effect of CD4^+^ T cells and CD8^+^ T cells on the pathological development and progression of PD by knocking the mice separately [76], and found that CD4^+^ T cells exacerbated PD pathological progression by possibly increasing the secretion of pro-inflammatory factors and promoting dopaminergic neuronal toxicity by activating the apoptotic signal Fas- Fas Ligand (FasL) [77]. However, the concept that all CD4^+^ T cells are considered to cause dopaminergic neurons damage is inaccurate. Further division of CD4^+^ T cells by function can be categorized into pro-inflammatory Th1 and Th17 cells and anti-inflammatory Th2 and Treg cells (Fig. 2). Among them, Treg cells are protective factors in the progression of the disease. Increasing the frequency of Treg cells through adoption, induction, and other methods can significantly improve the level of inflammation in the PD brain and alleviate the pathological process of PD [78]. In the study by Williams et al. [73], when mice were injected with adeno-associated virus 2 (AAV2)-synaptophysin (SYN) 4 weeks after the detection of T cell activation and corresponsive factors expression, it was found that the expression of transcription factors T-bet and Foxp3, cytokines interferon gamma (IFN-γ), and IL-10 in each of Th1 and Treg cells showed a significant increase, which indicated that both Th1 and Treg cell activation levels in the early stage of PD development showed an increase. This suggests that Treg cells may regulate the inflammatory response in the early stage of the disease, but the regulatory role of Treg cells on inflammation in the PD brain is gradually reduced/suppressed with the development of the disease, which eventually leads to a change in the ratio of pro-inflammatory cells to anti-inflammatory cells, resulting in an imbalance in the immune balance of the body and exacerbating the development of PD. This phenomenon may be related to the suppression of gamma-delta T (γδ T) cells. The frequency of γδ T cells in peripheral blood of PD patients is significantly reduced, and γδ T activated by IL-23 can directly inhibit the differentiation and function of Treg cells through the induction of heat-sensitive mediators or humoral factors with paracrine activity [79–81] (Fig. 2).

The cause of T-cell immune dysregulation in PD may also be related to dopamine release from dopaminergic neurons. Dopamine receptors (DR) are expressed not only in neurons but also in immune cells, thus possessing the ability to regulate immune functions, for instance, cell differentiation, cytokine release, and cytotoxicity [82–85]. Dopamine contributes to the differentiation of CD4^+^ T cells towards Th1 and Th17 by interacting with dopamine D3 receptor (DRD3) on their surface, increasing the number of pro-inflammatory T cells. At the same time, the combination of dopamine and dopamine D1 receptor (DRD1) inhibits the function of Treg cells, then causing dysregulation of immune regulation of the infiltrating brain T cell population [86]. In addition, abnormal alpha-synuclein in PD also affects the number and function of T cells, thereby triggering severe neurodegeneration [87]. Nitrated alpha-synuclein (N-α-synuclein) can disrupt immune tolerance and activate T cells in the periphery by diverting lymphoid tissue [88]. It was noted that using N-α-synuclein as an immunogen-induced effector T cell exacerbated microglia activation, thereby amplifying neuroinflammation and neurodegeneration [87–89]. Further investigation revealed that N-α-synuclein-stimulated T cells tend to differentiate more toward Th1 and Th17 phenotypes with pro-inflammatory cytokine release while suppressing Treg cell function [89].

In the pathological process of PD, T cells can be affected by abnormal α-synuclein, DA neurons, and thus immune response, and also interact with other immune cells in the internal environment to further influence the disease progression. Overall, T cells play an irreplaceable role in the pathological development and course of PD.

## Interactions between microglia and T cells and the related subtypes in PD

Microglia and T cells are linked in terms of activation and function. All of them interact with each other through the secretion of cytokines and chemokines, which have significant effects on the pathological state of PD. Next, we discuss the interactions between microglia and T cells and their related subtypes separately on neuronal damage and protection, and further investigate the interactions between microglia and T cells and their related subtypes in PD.

## The risk of neuronal damage from interactions between microglia and T cell-related subtypes in PD

Under the normal physiological state, immune cell activation maintains a stable dynamic balance. The immune cells and their related subtypes regulate each other to enable the clearance of abnormal substances to maintain the microenvironment homeostasis. However, there is the phenomenon of cytokine storm caused by excessive activation of immune cells in the PD brain, which indicates that the regulation of homeostasis between immune cells in a normal physiological state is faulty. The BBB is a vital tissue for maintaining the balance of the brain microenvironment. It effectively prevents peripheral immune cells, foreign invasive viruses, and neurotoxic substances in the blood from entering the brain and expels metabolic waste from the CNS out of the brain. However, the BBB has been shown to be severely damaged in PD brains, giving the opportunity for peripheral immune cells to infiltrate the brain parenchyma.

Following the alteration of the BBB permeability, immune cells in the peripheral circulation have the opportunity to infiltrate the brain parenchyma. Inflammatory factors as TNF-α and IL-1β released by microglia enhance the expression of cell adhesion molecules (intercellular adhesion molecule 1) and vascular cell adhesion molecules (vascular cell adhesion molecule 1) on vascular endothelial cells, further promoting the infiltration of peripheral immune cells into the brain parenchyma [38, 90, 91]. T cells infiltrating the brain parenchyma are induced by activated microglia to form pro-inflammatory types of T cell inflammation and through the release of inflammatory factors, exacerbating the extent of the BBB damage in PD and increasing the number of T cells infiltrating the brain [92]. These interactions lead to damage the BBB. In addition, the expression of MHC-II-like molecules on the surface of microglia activate T cells, and the infiltrating T cells simultaneously induce microglia to express MHC-II-like molecules, thus further deepening the interaction between these two cells [93, 94]. This has clarified the damaging behavior of the interactions on this BBB.

**Fig. 3:**
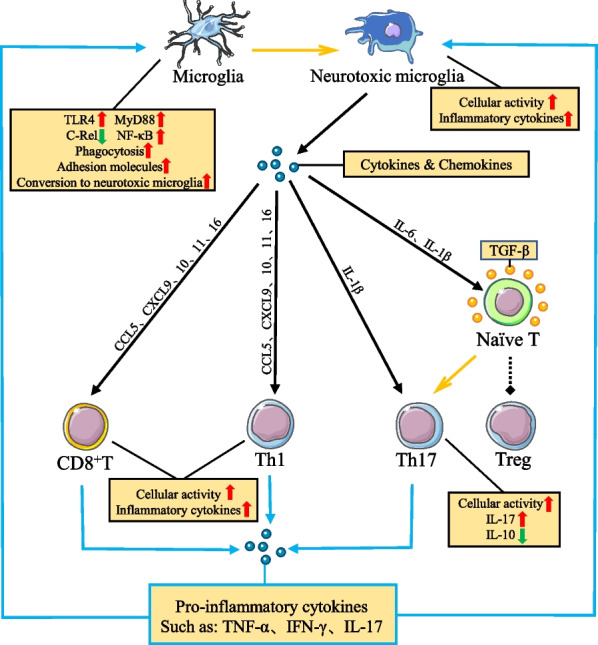
Microglia and T-cell interactions further amplify the inflammatory response to PD. The interaction of PD microglia and T cells amplifies the inflammatory response. In the presence of TGF-β in the microenvironment, IL-6 and IL-1β released from neurotoxic microglia act on naïve T cells had to induce their differentiation to Th17 cells and inhibit their differentiation to Treg cells, respectively. The chemokines CXCL9, CXCL10, CXCL11, CCL5, and CXCL16 released by neurotoxic microglia can bind to CD8^+^ T cells and CXCR3, CCR5 and CXCR6 on the surface of Th1 cells to further increase cell activation and release of inflammatory cytokines. Notably, inflammatory cytokines released from T cells can promote microglial activation and function. At the same time, microglia respond to inflammatory cytokines and increase their cellular activation by releasing cytokines and chemokines. Overall, the interaction between neurotoxic microglia and pro-inflammatory types of T cells in PD further amplifies the inflammation of the microenvironment and exacerbates neuronal damage

Neurotoxic microglia, as one of the primary forces for clearing abnormal substances from the brain, actively kill pathogenic substances by releasing various cytokines and chemokines, and recruit other immune cells to join the process. Among the cytokines, neurotoxic microglia promote and regulate the differentiation and function of Th17 cells by releasing inflammatory cytokines, while suppressing the differentiation of Treg cells, and TGF-β is an essential factor in the differentiation of naïve T to Th17 and Treg [95] (Fig. 3). When conditions of TGF-β factors are present in the environment, IL-6 and IL-1β released from neurotoxic microglia induce differentiation of naïve T cells infiltrating into the brain parenchyma to pro-inflammatory Th17 cells [96–99]. This is basically because IL-6 determines the fate of naïve T differentiation to Th17 cells by activating the transcription factor STAT3, while IL-1β inhibits the differentiation of naïve T cells to Treg cells. In addition, IL-1β increases the activity of Th17 and the release of the inflammatory cytokine IL-17 and decreases the secretion of the anti-inflammatory factor IL-10 [100] (Fig. 3).

Cytokines released from microglia can also act on dopaminergic neurons, causing their surface MHC-I-like molecule expression to be recognized and killed by CD8^+^ T cells infiltrating into the brain parenchyma, thereby accelerating the rate of dopaminergic neuron loss in PD (Fig. 2).

Chemokines play a significant role in the communication between neurons and immune cells, and they have chemotactic activity on immune cells and affect immune cell proliferation, cytokine secretion, and phagocytosis [101, 102]. In PD, the chemokines (C-X-C motif) ligand (CXCL) 9, CXCL10, CXCL11, CCL5, and CXCL16 released by neurotoxic microglia can increase their activation and functional expression by binding to C-X-C motif receptor (CXCR) 3, C–C motif receptor (CCR) 5, and CXCR6 chemokine receptors on the surface of T cells [103, 104] (Fig. 3). It was noted that inhibition of CXCR3 and CCR5 activation on T cells could reduce the number of CD3^+^ T cell infiltration in the substantia nigra [105, 106]. Besides, inhibition of CXCR3 activation reduced the production of IFN-γ activated T cells while protecting against lethal CD8^+^ T cell-mediated tissue damage [107, 108]. In contrast, inhibition of CCR5 activation polarized activated T cells toward Th2 cells and secreted anti-inflammatory factors such as IL-4 and IL-10, while decreasing IL-17 release from Th17 cells and alleviating neuronal loss. By comparison, CXCL16 released from microglia through CXCR6 receptors can drive Th1 and T-cytotoxic (Tc)1 cell migration and directly activate the NF-κB pathway in Th1 cells, increasing the expression of pro-inflammatory genes and enhancing T cell-mediated inflammatory responses [109–111].

Although CD8^+^ T cells can directly kill and release IFN-γ inflammatory factors to cause neurons death, CD4^+^ T cells are considered the major players in accelerating the PD processes. In particular, Th1 and Th17 cells promote the conversion of microglia to neurotoxic microglia and increase cellular functions (Fig. 3). Th1 cells increase the activity of the NF-κB pathway in microglia by releasing IFN-γ and TNF-α, respectively, increasing the expression of toll-like receptor 4 (TLR4) and myeloid differentiation primary response 88 (MyD88) in microglia and inhibiting the function of the c-Rel molecule. Simultaneously IFN-γ could further enhance the release of neurotoxic microglia chemokines [112–115]. On the other hand, Th17 enhanced the expression of adhesion molecules on microglia and the response to lipopolysaccharide (LPS) stimulation through the release of IL-17 [92, 116]. The effect of these two T cell subtypes on microglia increased the number of neurotoxic microglia and the release of pro-inflammatory cytokines (TNF-α, IL-1β, IL-6). It exacerbates oxidative stress and further disrupts the brain microenvironment homeostasis, thus accelerating neuronal loss [117] (Fig. 3). In addition to causing dopaminergic neurons damage by inducing the release of inflammatory factors from microglia, Th1 cells can also enhance the phagocytic capacity of microglia by upregulating the expression of transcripts of macrophage c-mer tyrosine kinase (MerTK), a receptor associated with phagocytosis, in microglia through the release of effector molecules, thus exacerbating the damage to dopaminergic neurons [115]. Enhanced phagocytosis of dopaminergic neurons by microglia during inflammation contributes to the pathological process of PD, which may be one of the reasons for the loss of dopaminergic neurons in PD [118–120].

Collectively, this evidence suggests that interactions between microglia and T cells in PD act synergistically to cause neuronal injury. The interactions between cells alter the brain homeostasis toward a pro-inflammatory state, promoting the number of neurotoxic microglia, Th1, and Th17, and increasing the release of corresponding inflammatory cytokines. At the same time, the interaction between microglia and T cells increases the phagocytic capacity of microglia. It allows CD8^+^ T cells to recognize dopaminergic neurons, thus accelerating the rate and extent of dopamine neuron damage, which in turn exacerbates the pathological state of PD.

## Protection of neurons by interactions between microglia and T cell-related subtypes in PD

Concerning PD treatment, inducing microglia to convert to a neuroprotective state and increasing their function can effectively slow neuronal death in PD [62, 69, 121, 122]. At the same time, Th2 and Treg cells in the CD4^+^ T cell subpopulation could also slow down the loss of dopaminergic neurons by increasing their number and functional expression. Further investigation revealed that significant microglia–T cell interactions in PD have an essential role in the remission and treatment of PD. Modulating neuronal damage resulting from imbalances in regulating environmental homeostasis in the brain by targeting the interaction between microglia and T cells could be a new starting point for treating PD.

**Fig. 4:**
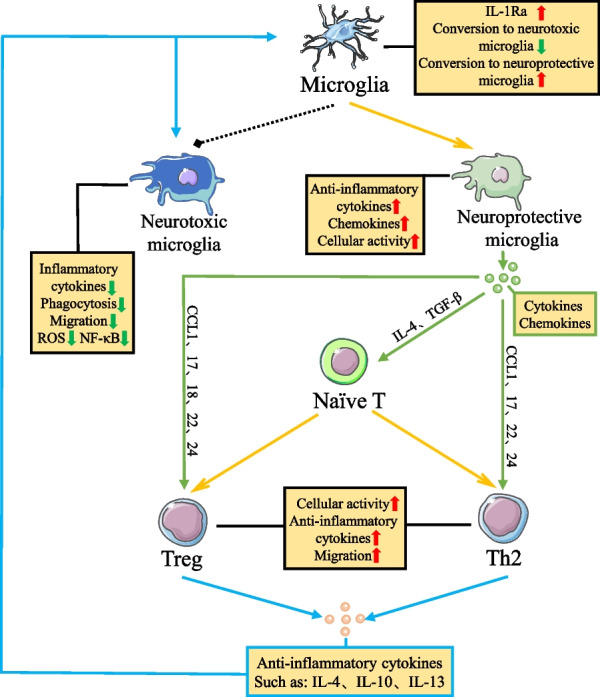
Microglia and T cells reciprocally regulate inflammation in PD. Neuroprotective microglia can induce the differentiation of naïve T cells to Th2 and Treg cells by secreting cytokines IL-4 and TGF-β. Meanwhile, chemokines CCL1, CCL17, CCL22, CCL24, and CCL18 released from neuroprotective microglia can bind to CCR4, CCR8, and PITPNM3 on the surface of Th2 and Treg cells, further increasing cell activation, anti-inflammatory cytokine release, and migration. Notably, anti-inflammatory cytokines released by Th2 and Treg cells can increase the number of neuroprotective microglia and inhibit the function of neurotoxic microglia. In conclusion, the interaction between neuroprotective microglia and anti-inflammatory T cell subtypes in PD increases the number and function of anti-inflammatory-type cells. At the same time, cellular interactions reduced the number and function of pro-inflammatory-type cells, thereby regulating the inflammatory state in the microenvironment and further protecting neurons from damage

First, how do neuroprotective microglia affect T-related subtypes of cells? Among cytokines, inflammation-suppressing cytokines such as IL-4, IL-10, IL-13, and TGF-β released from neuroprotective microglia in the normal physiological state can inhibit the production of pro-inflammatory cytokines such as IL-6 and TNF-α [123–125]. Meanwhile, IL-4 and TGF-β released from microglia are important cytokines that induce the differentiation of naïve T cells to Th2 and Treg cells, respectively, and have a significant impact on the developmental and functional regulation between T cells and their subpopulations (Fig. 4). Among the chemokines, neuroprotective microglia can induce T cell differentiation of Th2 and Treg cells and enhance cell function by releasing chemokines CCL1, CCL17, CCL22, and CCL24 that bind to T cell surface CCR4 and CCR8, thereby regulating the inflammatory state in the brain to protect neurons from death [103, 104] (Fig. 4). Moreover, CCL18 released from neuroprotective microglia, can block the recruitment of T cells in tumors, reduce the suppressive effect of Treg cells, and regulate the immune response by inhibiting the CCL18-PITPNM3 signaling pathway in tumor-related research [102, 126–129]. The role of CCL18 chemokine in PD is not clear. Considering the co-immunomodulatory role that Treg cells exhibit in PD and neoplastic disease, whether increasing the secretion of neuroprotective microglia CCL18 in PD can increase the number and function of Treg cells at the site of inflammation and thus regulate the inflammatory state needs to be verified by subsequent studies.

Next, how do infiltrating inflammatory T-cell affect microglia? The T cell that infiltrates the brain also exerts critical regulatory effects on inflammation through interactions with microglia. Among them, Th2 and Treg cells can induce the conversion of microglia to neuroprotective microglia and release anti-inflammatory cytokines and neurotrophic factors by releasing anti-inflammatory cytokines while reducing the number of neurotoxic microglia and the expression of related functions (Fig. 4). It was shown that microglia responding to IL-4 and IL-10 induce microglial conversion to neuroprotective microglia by increasing the activity of Janus kinase (JAK) signal transducer and activator of transcription (STAT) 6 pathway and decreasing the activity of JAK–STAT3 pathway in microglia, respectively. This decrease in neurotoxic microglia is due to the inhibition of STAT1 phosphorylation by IL-10, which in turn leads to a decrease in the activity of the NF-κB pathway [130–135]. Meanwhile, IL-4 and IL-13 released from Th2 cells induced IL-1 receptor antagonist (IL-1Ra) expression blocking the induction of IL-1β into microglia, further reducing the number of neurotoxic microglia and the release of inflammatory cytokines and chemokines [136, 137]. In addition, infiltrating Treg cells also inhibit the activation of microglia by nitrocellularized α-synuclein and regulate the state of neurotoxic microglia and the release of inflammatory factors by reducing the migration, phagocytosis, reactive oxygen species (ROS) production, and NF-κB activation of neurotoxic microglia [87, 89, 138]. Notably, Treg can also reduce the number of neurotoxic microglia by inducing apoptosis, thus protecting neurons from dopaminergic neuronal damage caused by excessive activation of neurotoxic microglia [117].

Overall, this evidence suggests that the interaction between microglia and T cells increases the number and function of neuroprotective microglia, Th2, and Treg cells and effectively reduces the conversion of microglia to neurotoxic microglia, decreases the activation of pro-inflammatory type T cells, thus decreasing the level of inflammatory cytokine secretion in the brain and regulating the dysregulation of internal environmental homeostasis, which in turn protects against damage to dopaminergic neurons in PD and slows down the pathological symptoms and disease process.

## A preliminary survey of the dysregulation of protective effects by microglia–T cell interactions in PD

Previously, we described the relevant pathways of neuronal damage by inflammatory factor storms mediated by immune cells in PD. The relevant role of microglia and T cell and their related subtype interactions for damage and protection in PD has also been described. However, the regulatory role of microglia and T cell-related subtypes interactions on inflammation seems to be diminished or to have failed in PD. The possible reasons are: (1) the negative feedback mechanism of age-related neuroprotective microglia regulating inflammatory state may be defective. The increase in pro-inflammatory levels of microglia with age is accompanied by a "dystrophic" phenomenon of denuclearization and process fragmentation [139, 140]. This causes a decrease in receptor expression and cytokine secretion converting microglia to neuroprotective microglia, resulting in a decrease in the number of neuroprotective microglia and, thus, a significant reduction in the regulation of neurotoxic microglia [141]. (2) The microenvironment in the brain promotes the differentiation and functional expression of the inflammatory types of cells, further contributing to immune dysregulation. T cells infiltrated into the brain were induced by pro-inflammatory microglia, dopamine secreted by neurons, the internal environment of the brain and other factors, and the activation state and function of pro-inflammatory Tc, Th1, and Th17 cells were further enhanced, while the function of anti-inflammatory Th2 and Treg was suppressed. At the same time, naïve T cells were also induced to polarize toward pro-inflammatory types of Th1 and Th17, which increased the number of pro-inflammatory cell types, further promoting inflammation in the cerebral environment and weakening the regulatory effect of anti-inflammatory cell types. In this process, the increased inflammatory state of the brain environment promotes the conversion of microglia to neurotoxic microglia. It releases inflammatory cytokines and neurotoxic mediators to aggravate neuronal damage further, resulting in a vicious cycle between dying neurons and acute inflammation [142, 143].

## A potential new model for studying the cellular interactions in PD

In previous studies on the pathogenesis of PD, experimental investigations were often conducted using model animals and cells. These models have had significant results, and the pathological mechanisms associated with PD have been further explained. However, some of the limitations of the above models include epigenetic differences between species, leading to findings that do not accurately describe the pathological process in human PD patients [144]. Although data were obtained in some studies by taking postmortem brain tissue samples from human PD patients, it should be taken into account that this data only reflects the pathological changes in the brains of patients with end-stage PD [145]. The development of PD is a slow process, and the presence of primary microglia and dopamine neurons that are more difficult to acquire and fail to proliferate has created some difficulties in exploring the development of this disease in the brain of PD patients. Along with the rapid development of induced pluripotent stem cells (iPSC) technology, researchers have used a variety of terminally differentiated cells from PD patients to induce them to become midbrain dopamine neurons, and microglia, by iPSC technology and further characterized them using gene sequencing, calcium imaging, and electrophysiological methods [146–149]. iPSC-induced cells were found to be highly consistent with dopamine neurons and microglia in the brains of human PD patients. This model allows for a better investigation of the pathological effects of genetic mutations and other factors in sporadic PD patients [144, 150]. Currently, iPSC models have been used to study PD pathogenesis. In contrast, in the study on PD neuroinflammation, it was found that Th17 cells act on iPSC-induced neurons by releasing IL-17 to increase their surface IL-17 receptor expression and NF-κB activation-induced neuronal death [151]. In recent years, as iPSC technology has become more mature, the establishment of organoid models has also been rapidly developed, enabling the derivation of in vitro 2D models to 3D models that better assess the spatial effects of neurons and other outcomes. However, the current midbrain organoid system is mainly derived from neuroepithelial stem cells, resulting in a lack of microglia in this model, making its use in PD neuroinflammation problematic [152]. However, a recent study has successfully integrated human functional microglia into the midbrain organoid system and demonstrated the feasibility and stability of this approach by examining the gene expression, functional changes, and communication ability of microglia in this model [152]. The use of this microglia-embedded midbrain organoid model to study the interaction between microglia and T cells in PD on the pathological development of PD is expected to provide a more detailed and accurate assessment of the impact of microglia–T cell interaction on the progressive pathological process of human PD patients.

## Conclusion

This article illustrates that interactions between microglia and T cells and their related subtypes exhibit a significant role in the onset and course of PD. Overall, the interactions among neurotoxic microglia, Tc, Th1, and Th17 cells in PD raise the level of inflammation in the microenvironment and elevate the activation and function of pro-inflammatory cells, amplifying the effects of immune cells on neuronal damage, thus exacerbating the pathological condition of PD and accelerating the disease process. Meanwhile, there is a complex interplay between neuroprotective microglia, Th2, and Treg cells in regulating the activation and function of pro-inflammatory cells, and controlling the inflammatory state of the internal environment, furthermore reducing neuronal damage. These cells could protect neurons from damage in PD by potentiating the communications between microglia and T cells and their related subtypes in PD to regulate the inflammatory cytokine storm caused by excessive activation of immune cells. This review also focuses on the CCL18–PITPNM3 signaling pathway and γδ T cells, which show great potential in immune regulation and may serve as potential targets for PD therapy, providing new ideas for the treatment of PD. Meanwhile, a potential new model for exploring the effect of microglia–T cell interactions on the development of PD pathology is provided.

## Data Availability

Not applicable.

## Abbreviations

PD: Parkinson’s disease
Th: T-helper
CCL: C–C motif ligand
PITPNM3: Phosphatidylinositol transfer protein 3
CNS: Central nervous system
TNFR: Tumor necrosis factor receptor
TNF-α: Tumor necrosis factor-alpha
NF-κB: Nuclear factor-kappa B
BBB: Blood–brain barrier
CD: Cluster of differentiation
TGF-β: Transforming growth factor-beta
IL: Interleukin
MHC: Major histocompatibility complex
FasL: Fas Ligand
AAV2: Adeno-associated virus 2
SYN: Synaptophysin
IFN-γ: Cytokines interferon gamma
γδ T: Gamma-delta T
DR: Dopamine receptors
DRD3: Dopamine D3 receptor
DRD1: Dopamine D1 receptor
CXCL: C-X-C motif ligand
CXCR: C-X-C motif receptor
CCR: C–C motif receptor
Tc: T-cytotoxic
TLR4: Toll-like receptor 4
MyD88: Myeloid differentiation primary response
LPS: Lipopolysaccharide
MerTK: Macrophage c-mer tyrosine kinase
JAK: Janus kinase
STAT: Signal transducer and activator of transcription
IL-1Ra: IL-1 receptor antagonist
ROS: Reactive oxygen species
IPSC: Induced pluripotent stem cells
NLRP3: NOD-like receptor family, pyrin domain containing 3
N-α-synuclein: Nitrated alpha-synuclein
